# How the Replication and Transcription Complex Functions in Jumping Transcription of SARS-CoV-2

**DOI:** 10.3389/fgene.2022.904513

**Published:** 2022-05-30

**Authors:** Jianguang Liang, Jinsong Shi, Shunmei Chen, Guangyou Duan, Fan Yang, Zhi Cheng, Xin Li, Jishou Ruan, Dong Mi, Shan Gao

**Affiliations:** ^1^ School of Pharmacy, Changzhou University, Changzhou, China; ^2^ National Clinical Research Center of Kidney Disease, Jinling Hospital, Nanjing University School of Medicine, Nanjing, China; ^3^ Yunnan Key Laboratory of Stem Cell and Regenerative Medicine, Biomedical Engineering Research Center, Kunming Medical University, Kunming, China; ^4^ School of Life Sciences, Qilu Normal University, Jinan, China; ^5^ College of Life Sciences, Nankai University, Tianjin, China; ^6^ School of Mathematical Sciences, Nankai University, Tianjin, China; ^7^ Department of Clinical Laboratory, Affiliated Maternity Hospital, Nankai University, Tianjin, China

**Keywords:** coronavirus, RNA methylation, nanopore, TRS hairpin, METTL3

## Abstract

**Background:** Coronavirus disease 2019 (COVID-19) is caused by severe acute respiratory syndrome coronavirus 2 (SARS-CoV-2). Although unprecedented efforts are underway to develop therapeutic strategies against this disease, scientists have acquired only a little knowledge regarding the structures and functions of the CoV replication and transcription complex (RTC). Ascertaining all the RTC components and the arrangement of them is an indispensably step for the eventual determination of its global structure, leading to completely understanding all of its functions at the molecular level.

**Results:** The main results include: 1) hairpins containing the canonical and non-canonical NSP15 cleavage motifs are canonical and non-canonical transcription regulatory sequence (TRS) hairpins; 2) TRS hairpins can be used to identify recombination regions in CoV genomes; 3) RNA methylation participates in the determination of the local RNA structures in CoVs by affecting the formation of base pairing; and 4) The eventual determination of the CoV RTC global structure needs to consider METTL3 in the experimental design.

**Conclusions:** In the present study, we proposed the theoretical arrangement of NSP12-15 and METTL3 in the global RTC structure and constructed a model to answer how the RTC functions in the jumping transcription of CoVs. As the most important finding, TRS hairpins were reported for the first time to interpret NSP15 cleavage, RNA methylation of CoVs and their association at the molecular level. Our findings enrich fundamental knowledge in the field of gene expression and its regulation, providing a crucial basis for future studies.

## Introduction

Coronavirus disease 2019 (COVID-19) is caused by severe acute respiratory syndrome coronavirus 2 (SARS-CoV-2) (Li et al., 2020) (Duan et al., 2020) with a genome of ∼30 kb (Jiayuan et al., 2020). By reanalyzing public data (Kim et al., 2020a), we determined that a SARS-CoV-2 genome has 12 genes, which are *spike (S)*, *envelope (E)*, *membrane (M)*, *nucleocapsid (N)*, and *ORF1a*, *1b*, *3a*, *6*, *7a*, *7b*, *8* and *10* (Li et al., 2021a). The *ORF1a* and *1b* genes encode 16 non-structural proteins (NSPs), named NSP1 through NSP16 (Silva et al., 2020), while the other 10 genes encode four structural proteins (S, E, M and N) and six accessory proteins (ORF3a, 6, 7a, 7b, 8 and 10). Among the above 26 proteins, NSP4-16 are highly conserved in all known CoVs and have been experimentally demonstrated or predicted to be critical enzymes in CoV RNA synthesis and modification (Denison et al., 2011), particularly including: NSP12, RNA-dependent RNA polymerase (RdRP) (Yan et al., 2020); NSP13, RNA helicase-ATPase (Hel); NSP14, RNA exoribonuclease (ExoN) and N7 methyltransferase (MTase); NSP15 endoribonuclease (EndoU) (Kim et al., 2020b); and NSP16, RNA 2′-O-MTase.

NSP1-16 assemble into a replication and transcription complex (RTC) (Yan et al., 2020). The basic function of the RTC is RNA synthesis: it synthesizes genomic RNAs (gRNAs) for replication or transcription of the *ORF1a*, *1b* genes, while it synthesizes subgenomic RNAs (sgRNAs) for jumping transcription of the other 10 genes (Kim et al., 2020a). In 1998, the “leader-to-body fusion” model (Sawicki et al., 1998) was proposed to explain the jumping transcription, however, the molecular basis of this model was unknown until our previous study in 2020 (Li et al., 2021a). In our previous study (Li et al., 2021a), we provided a molecular basis for the “leader-to-body fusion” model by identifying the cleavage sites of NSP15 and proposed a negative feedback model to explain the regulation of CoV replication and transcription. In addition, we revealed that the jumping transcription and recombination of CoVs share the same molecular mechanism (Li et al., 2021a), which causes rapid mutation and inevitably outbreaks of CoVs. These findings are vital for the further investigation of CoV transcription and recombination. However, there will be a long way to completely understand how the RTC functions in the jumping transcription at the molecular level.

For a complete understanding of CoV replication and transcription, particularly the jumping transcription, much research (Yan et al., 2020) (Kim et al., 2020b) (Hillen et al., 2020) has been conducted to determine the global structure of the SARS-CoV-2 RTC, since the outbreak of SARS-CoV-2 in 2019. Although some single protein structures (*e.g.*, NSP15 (Kim et al., 2020b)) and local structures of the RTC (i.e. NSP7&8&12&13 (Yan et al., 2020) and NSP7&8&12 (Hillen et al., 2020)) have been determined, the global structure and all components of RTC are still unknown. As the global structure of the CoV RTC cannot be determined by simple use any one of current methods (i.e., X-ray, NMR and Cryo-EM), ascertaining all the RTC components and the arrangement of them is an indispensably step for the eventual determination of its global structure, leading to completely understanding all of its functions at the molecular level. In the present study, we aimed to determine the theoretical arrangement of NSP12-16 in the global structure of the CoV RTC by comprehensive analysis of data from different sources, and to preliminarily elucidate how the RTC functions in the jumping transcription of CoVs at the molecular level.

## Results

### Jumping Transcription, TRS and NSP15 Cleavage Site

First, we provide a brief introduction to the jumping transcription of CoVs, the “leader-to-body fusion” model proposed in an early study (Sawicki et al., 1998) and its molecular basis proposed in our recent study (Li et al., 2021a). In the “leader-to-body fusion” model, the realization of jumping transcription requires transcription regulatory sequences (TRSs), which include leader TRSs (TRS-Ls) and body transcription regulatory sequences (TRS-Bs). Each CoV genome contains a TRS-L in the 5′ untranslated region (UTR) and several TRS-Bs located in the upstreams of genes except *ORF1a and 1b*. CoV replication and transcription require gRNAs(+) as templates for the synthesis of antisense genomic RNAs [gRNAs(-)] and antisense subgenomic RNAs [sgRNAs(-)] by RdRP. When RdRP pauses, as it crosses a TRS-B and switches the template to the TRS-L, sgRNAs(-) are formed through jumping transcription (also referred to as discontinuous transcription, polymerase jumping or template switching). Otherwise, RdRP reads gRNAs(+) continuously, without interruption, resulting in gRNAs(-). Thereafter, gRNAs(-) and sgRNAs(-) are used as templates to synthesize gRNAs(+) and sgRNAs(+), respectively; gRNAs(+) and sgRNAs(+) are used as templates for the translation of NSP1-16 and the other 10 proteins (S, E, M, N, and ORF3a, 6, 7a, 7b, 8 and 10), respectively. In our previous study (Li et al., 2021a), we provided a molecular basis for the “leader-to-body fusion” model by identifying the reverse complimentary sequences of TRS-Bs [denoted as TRS-Bs(-)] as the NSP15 cleavage sites, which actually functions in the regulation of CoV regulation. NSP15 cleaves gRNAs(-) and sgRNAs(-) at TRS-Bs(-). Then, the free 3′ ends (∼6 nt) of TRS-Bs(-) hybridize TRS-Ls to realize “leader-to-body fusion”. These findings associated the investigation of TRSs to that of NSP15 cleavage sites.

**FIGURE 1:**
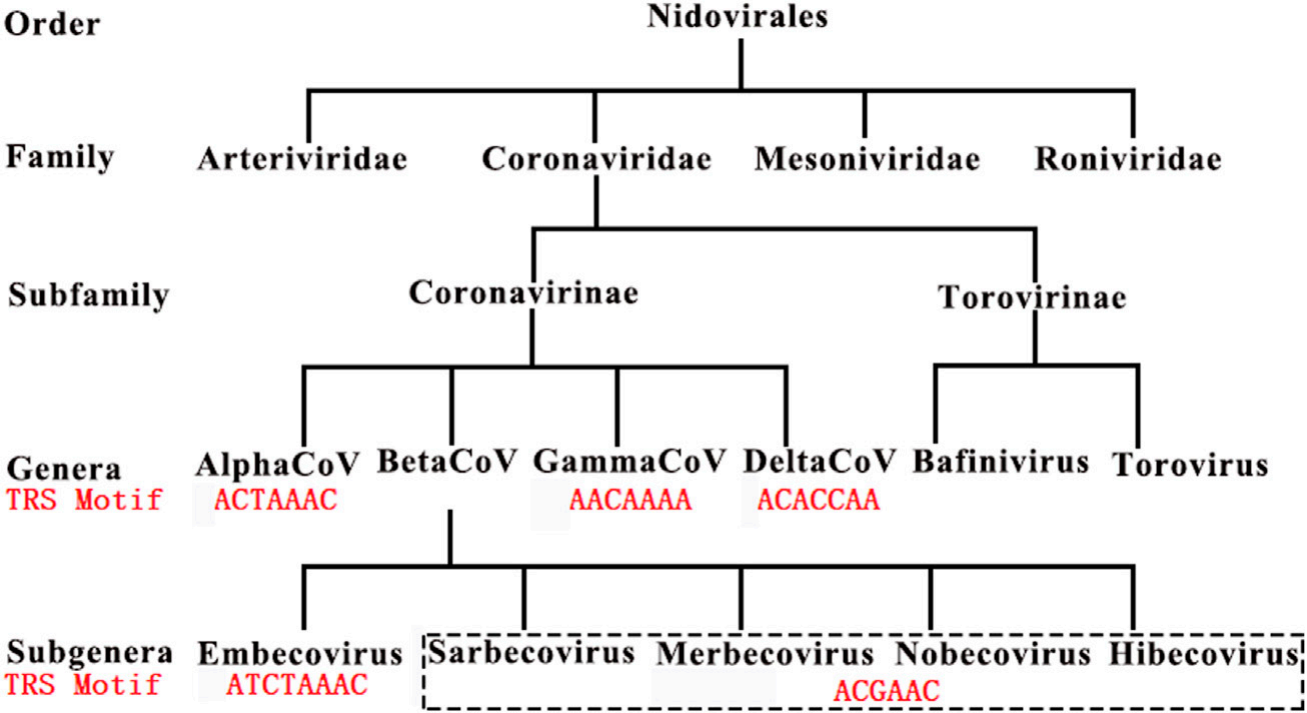
Canonical TRS motifs in Coronaviridae. *Embecovirus*, *Sarbecovirus*, *Merbecovirus*, *Nobecovirus* and *Hibecovirus* are also defined as *Betacoronavirus* subgroups A, B, C, D and E. SARS-CoV and SARS-CoV-2 belong to *Betacoronavirus* subgroup B. These canonical TRS motifs (in red color) of viruses in Coronaviridae have been reported in our previous study (Bei et al., 2022).

In our previous study (Bei et al., 2022), we made a generalization that a TRS motif is a (6∼8-nt long for CoVs) consensus sequence beginning with at least an adenosine residue (**A**), and enriched with A and followed by C, based on the analysis of 1,265 CoV genome sequences (**Materials and Methods**). We defined that the antisense sequence of a TRS motif as the motif of the corresponding NSP15 cleavage site (the NSP15 cleavage motif). For example, the canonical TRS motif of SARS-CoV-2 and the corresponding NSP15 cleavage motif are **A**CGAAC and GTTCG**T**, respectively. We defined the TRS motif in the TRS-L as the canonical TRS motif. Thus, the canonical TRS motif is unique to a CoV genome, while the TRS motifs in TRS-Bs can be canonical TRS motifs or non-canonical TRS motifs with little nucleotide (nt) differences. By these definitions, we determined canonical TRS motifs of all viruses in the order *Nidovirales* (Figure 1) and corrected some canonical TRS motifs reported in the previous studies. For instance, the canonical TRS motifs of mouse hepatitis virus (MHV), transmissible gastroenteritis virus (TGEV), canada goose coronavirus (Goose-CoV) and beluga whale coronavirus (BWCoV) were corrected from CTAAAC (Grossoehme et al., 2009), CTAAAC (Sola et al., 2005), CTTAACAAA (Papineau et al., 2019) and AAACA (Mihindukulasuriya et al., 2008) to **A**TCTAAAC, **A**CTAAAC, **A**ACAAAA and **A**ACAAAA, respectively. Canonical TRS motifs are highly conserved in *Alphacoronavirus*, *Gammacoronavirus*, *Deltacoronavirus* and *Betacoronavirus* genera except the subgroup A (Figure 1). *Betacoronavirus* subgroup A has the canonical TRS motif **A**TCTAAAC, which is different from **A**CGAAC in *Betacoronavirus* subgroup B, C, D and E. Different from *Betacoronavirus* subgroup B, *Betacoronavirus* subgroup A, C, D and E, *Alphacoronavirus*, *Gammacoronavirus* and *Deltacoronavirus* have non-canonical TRS motifs in the TRS-Bs of four structural genes (*S*, *E*, *M* and *N*), which were caused by mutations during evolution. These TRS motif mutations resulted in the attenuation of CoVs in *Betacoronavirus* subgroup A, D and E by down-regulating the transcription of CoV genes except *ORF1a* and *1b* (Li et al., 2021b). This confirmed that TRSs (Actually revealed as the NSP15 cleavage sites (Li et al., 2021a)) function in the regulation of CoV transcription (Yount et al., 2006). Furthermore, a previous study reported that the recognition of a TRS (Actually revealed as the NSP15 cleavage site (Li et al., 2021a)) is independent on its motif, but dependent on its context (Yount et al., 2006).

### NSP15 Cleavage, RNA Methylation and TRS Hairpin

A previous study (Kim et al., 2020a) reported that RNA methylation sites containing the “AAGAA-like” motif (including AAGAA and other A/G-rich sequences) are present throughout the SARS-CoV-2 genome, particularly enriched in genomic positions 28,500-29,500. This study used Nanopore RNA-seq (Xu et al., 2019), a direct RNA sequencing method, which can be used to measure RNA methylation at 1-nt resolution although it has a high error rate. By analyzing the Nanopore RNA-seq data, the previous study (Kim et al., 2020a) concluded that the methylated RNAs have shorter 3′ polyA tails than the unmethylated ones in SARS-CoV-2. Although the type of RNA methylation was unknown, the previous study (Kim et al., 2020a) proposed that the “AAGAA-like” motif associates with the lengths of 3′ polyA tails of gRNAs and sgRNAs. However, the previous study did not answer the following questions: 1) it was not explained that what functions the internal methylation sites have, as they are far from 3′ ends, thus unlikely to contribute to the lengths of 3′ polyA tails; and 2) the extremely high ratio between sense and antisense reads (Li et al., 2021a) may result from quick degradation of the antisense nascent RNAs due to their shorter 3′ polyA tails, however, the “AAGAA-like” motif occurs in both sense and antisense strands at a similar frequency. Notably, the previous study (Kim et al., 2020a) shouldn’t have neglected the analysis of the “AAGAA-like” motif on the antisense strand, since only very a few antisense reads from the Nanopore RNA-seq data were obtained for analysis. Therefore, we proposed that RNA methylation sites containing the “AAGAA-like” motif may have other biological functions and conducted further analysis.

**FIGURE 2:**
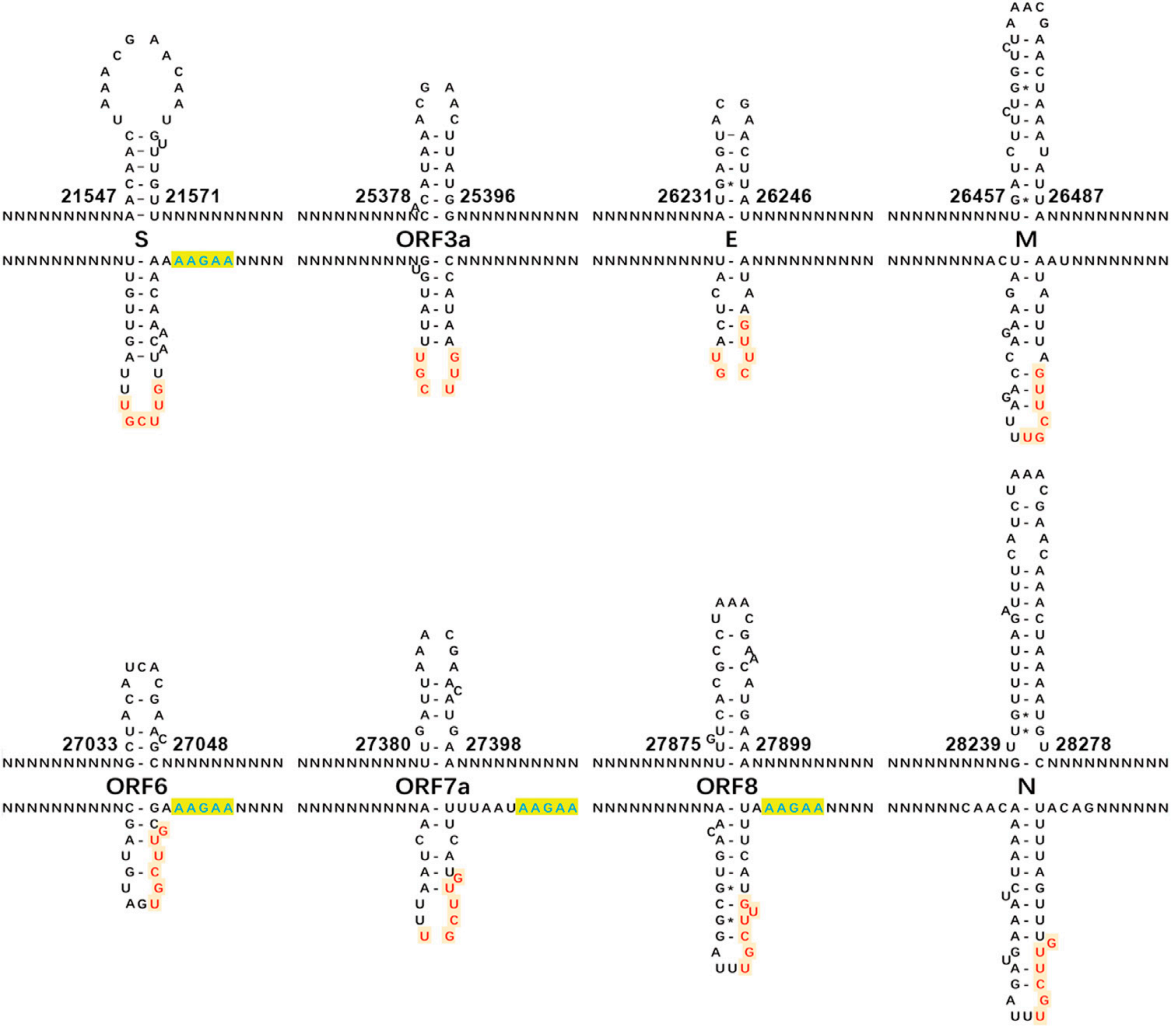
Canonical TRS hairpins in SARS-CoV-2. The canonical transcription regulatory sequence (TRS) motif ACGAAC is present in the upstreams of eight genes (*S*, *E*, *M*, *N*, and *ORF3a*, *6*, *7a* and *8*). Read on the antisense strands of the SARS-CoV-2 genome (GenBank: MN908947.3), “AAGAA” (in blue color) or “AAACH” (Supplementary Table S1) represents an RNA methylation site, while “GUUCGU” (in red color) represents a NSP15 cleavage site. The positions are the start and end positions of hairpins in the SARS-CoV-2 genome. NSP15 cleaves a single-strand RNA after U (indicated by arrows). In the present study, we defined: (1) the hairpins containing the canonical and non-canonical NSP15 cleavage sites are canonical and non-canonical TRS hairpins, respectively; and (2) the hairpins opposite to the TRS hairpins as the opposite TRS hairpins.

**FIGURE 3:**
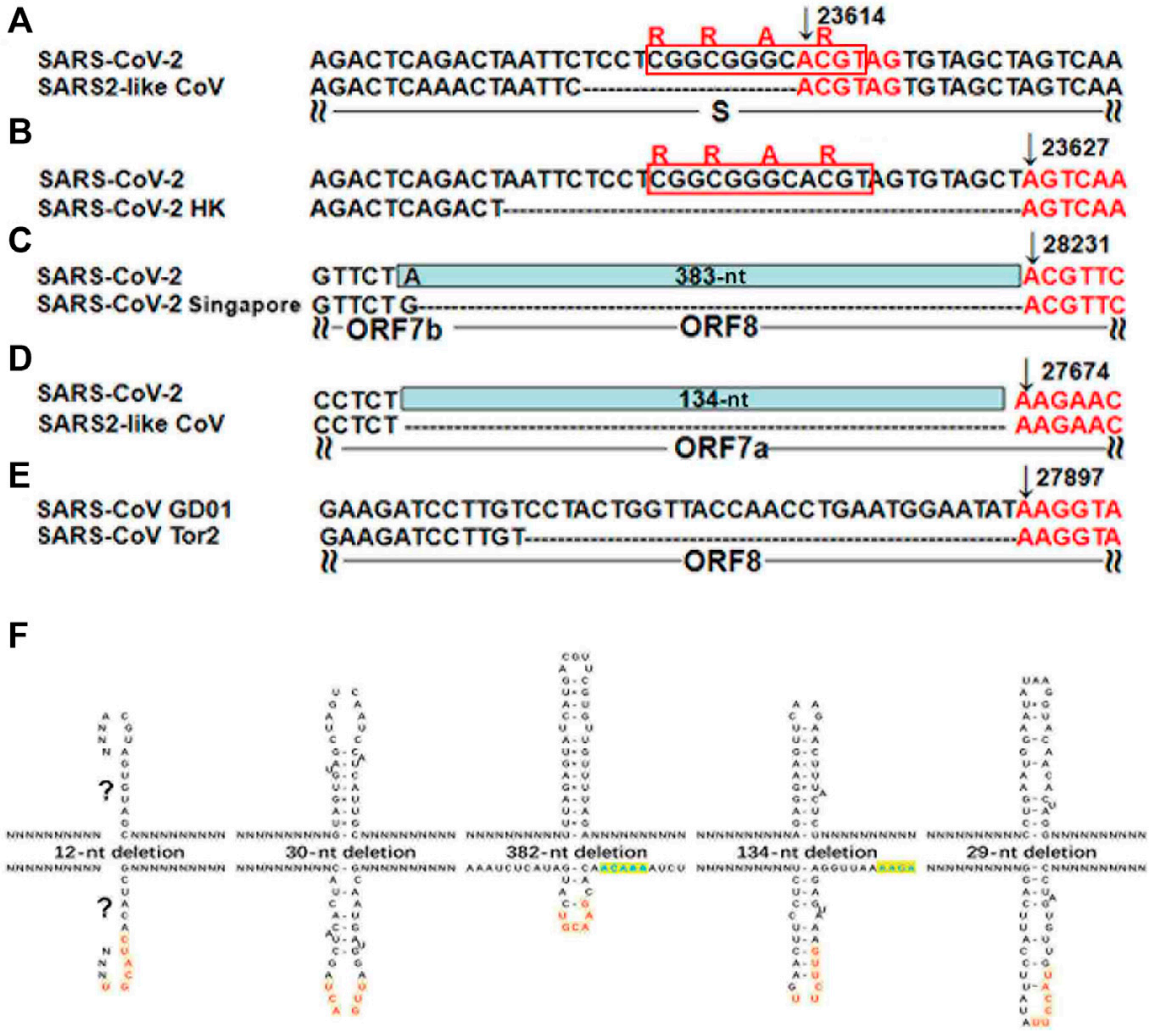
TRS hairpins in five recombination regions. **(A-E)** have already been published in our previous study (Li et al., 2021a). N represents any nt. All the positions were annotated on the SARS-CoV (GenBank: AY278489) or SARS-CoV-2 (GenBank: MN908947) genomes. **(A)**. The genome (GenBank: MN996532) of the SARS2-like CoV strain RaTG13 from bats is used to show the 12-nt deletion; **(B)**. The genome (GISAID: EPI_ISL_417443) of the SARS-CoV-2 strain Hongkong is used to show the 30-nt deletion; **(C)**. The genomes (GISAID: EPI_ISL_414378, EPI_ISL_414379 and EPI_ISL_414380) of three SARS-CoV-2 strains from Singapore are used to show the 382-nt deletion; **(D)**. The genome (GenBank: MT457390) of the mink SARS2-like CoV strain is used to show the 134-nt deletion; **(E)**. The genome (GenBank: AY274119) of the SARS-CoV strain Tor2 is used to show the 29-nt deletion . **(F)**. These recombinant events occurred at the non-canonical NSP15 breakpoints that also end with at least an uridine residue (“U”), due to the cleavage of the non-canonical TRS hairpins.

Different from the previous study (Kim et al., 2020a), our study focused on the analysis of the “AAGAA-like” motif on the antisense strand of the SARS-CoV-2 genome, particularly the association between the “AAGAA-like” motif and the TRS or corresponding NSP15 cleavage motifs. As a result, we discovered that the “AAGAA-like” motif co-occurred with the NSP15 cleavage motif GTTCG**T** of four genes (*S, ORF6, 7a* and *8*). In our previous study (Liu et al., 2018), complemented palindrome sequences in genomes of viruses in *Betacoronavirus* subgroup B have been investigated and most of them are semipalindromic or heteropalindromic. These complemented palindrome sequences containing A-rich and T-rich regions form hairpins. The “AAGAA-like” and GTTCG**T** motifs are located in the A-rich and T-rich regions. Thus, the association between the “AAGAA-like” and GTTCG**T** motifs was discovered by analysis of TRS hairpins of the four genes (Figure 2). For analysis of TRS hairpins, we defined: 1) hairpins containing the canonical and non-canonical NSP15 cleavage sites are canonical and non-canonical TRS hairpins, respectively; and 2) hairpins opposite to TRS hairpins are opposite TRS hairpins (Figure 2). However, the formation of opposite TRS hairpins is uncertain, as all complemented palindrome sequences forming the TRS and opposite TRS hairpins are asymmetric (semipalindromic or heteropalindromic). Among the 10 genes, eight (*S*, *E*, *M*, *N*, *ORF1a*, *1b*, *3a*, *6*, *7a*, and *8*) have canonical TRS hairpins and two (*ORF7b* and *10*) may have non-canonical TRS hairpins (Supplementary Table S1). Non-canonical TRS hairpins have been reported in seven common recombination regions in one of our previous studies (Li et al., 2021b) and identified in five recombination events (Figure 3) in another one of our previous studies (Li et al., 2021a). Therefore, TRS hairpins can be used to identify recombination regions in CoV genomes. NSP15 cleaves the canonical TRS hairpins of the seven genes at canonical breakpoints, whereas it cleaves the canonical TRS hairpin of *ORF3a* at an unexpected breakpoint “**GTTCGT**TTAT|N” (the NSP15 cleavage motif is underlined; the vertical line indicates the breakpoint and N represents any nt), rather than the end of the canonical NSP15 cleavage motif “**GTTCGT**|TTATN”. According to our definitions, “**GTTCGT**|TTATN” and “**GTTCGT**TTAT|N″ are canonical and non-canonical NSP15 breakpoints, respectively. The discovery of non-canonical TRS hairpins and non-canonical NSP15 breakpoints indicated that the recognition of NSP15 cleavage sites is structure-based rather than sequence-based.

### How RTC Functions in Jumping Transcription

**FIGURE 4:**
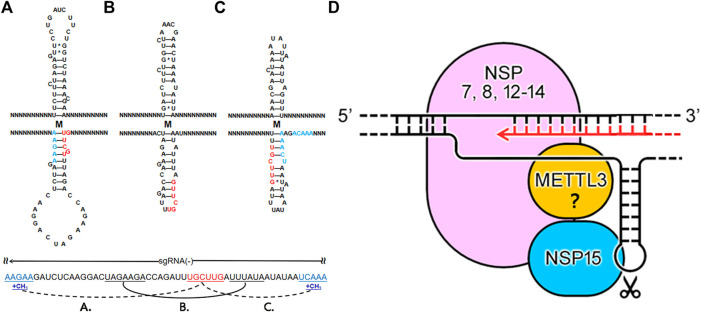
How RTC functions in jumping transcription. N represents any nt. Using the *M* gene of SARS-CoV-2 as an example, the first **(A)** and third **(C)** hairpins require the “AAGAA-like” or AAACH motifs involved in the base pairing. RNA methylation of sequences containing the “AAGAA-like” or AAACH (in blue color) motifs is not in favour of base pairing, ensuring the formation of a unique TRS hairpin **(B)** containing a NSP15 cleavage site in the loop **(D)** 5′-3′ represents the strand of the SARS-CoV-2 genome. NSP12-14 form the main structure of the RTC; NSP7 and NSP8, acting as the cofactors of NSP12, may be also included in the main structure of the RTC (Yan et al., 2020); NSP15 and METTL3 are coupled with the main structure. The RTC processes the double-strand RNAs (dsRNAs) and single-strand RNAs (ssRNAs) in two situations. Nascent RNAs are synthesized in one route using unwound ssRNAs(+) or ssRNAs(-) as templates. In the other route, ssRNAs(-) can be uncleaved or cleaved for jumping transcription or degraded, which is regulated by a negative feedback mechanism (Li et al., 2021a). NSP15 cleaves a ssRNA in a small loop in the second route.

Since several A-rich and T-rich regions are alternatively present around each NSP15 cleavage site, many hypothetical TRS hairpins (Figure 4A–C) containing the NSP15 cleavage site can form. Thus, to investigate if a unique TRS hairpin can be formed, we further analyzed the association between the “AAGAA-like” and GTTCGT motifs in all possible TRS hairpins of the eight genes (Supplementary Table S1) using 1,265 CoV genome sequences (Materials and Methods), leading to discovery of the association between RNA methylation and NSP15 cleavage. Here, we illustrate how the association was discovered, using the *M* gene of SARS-CoV-2 as an example (Figure 4). The minimum free energies (MFEs) of three possible TRS hairpins in the *M* gene were estimated as -2.50, -4.00 and -4.90 kcal/mol (Materials and Methods). Although the third hairpin (Figure 4C) is the most stable one, the difference of MFEs between the second (Figure 4B) and third hairpins is marginal. The first (Figure 4A) and third hairpins require the “AAGAA-like” and AAACH (Detailed later) motifs involved in the base pairing, respectively. However, RNA methylation (*e.g.*, m6A) of these motifs is not in favour of base pairing in the first and third hairpins. Thus, only the second hairpin was able to form. We proposed that RNA methylation participates in the determination of the local RNA structures in CoVs by affecting the formation of base pairing. RNA methylation of sequences containing the “AAGAA-like” or AAACH motifs significantly reduces the possibility of formation of many hairpins, ensuring the formation of a unique TRS hairpin (Figure 4B) in all likelihood. In the unique TRS hairpin, the NSP15 cleavage site exposes in a small loop, which facilitates the contacts of NSP15, while the loop of the opposite TRS hairpin may not contain uridine residues for NSP15 cleavage. The structure of this small loop can be used to explain the results of mutation experiments in a previous study (Yount et al., 2006) that the recognition of a TRS (Actually revealed as the NSP15 cleavage site (Li et al., 2021a)) is independent on its motif, but dependent on its context. The TRS hairpin can be used to explain the discovery that the recognition of NSP15 cleavage sites is structure-based (TRS hairpin) rather than sequence-based (NSP15 cleavage motif). The above results indicated that TRS hairpins in nascent gRNAs(-) are indispensable for the functions of the RTC in jumping transcription (Figure 4D).

The following topic is which enzyme is responsible for the internal methylation of CoV RNAs, which is supposed to be done before the NSP15 cleavage for jumping transcription. A recent study reported that NSP14 (no structure data available) and NSP10&16 (PDB: 7BQ7), as N7 and 2′-O-MTase respectively (Introduction), are crucial for RNA cap formation (Krafcikova et al., 2020). This suggested that NSP14 and NSP10&16 are unlikely to function in the internal methylation of CoV RNAs. Although the previous study excluded METTL3-mediated RNA (m6A) methylation for lack of the canonical motif RR**A**CH (R and H represent A/G and A/C/T, respectively) (Kim et al., 2020a), we still found many internal methylation sites containing the AAACH motif in the SARS-CoV-2 genome by reanalyzing the Nanopore RNA-seq data. Notable instances include “ag**T**tt” (AAACT on the antisense strand) at the positions 29408 and 29444 (corresponding to the underlined capital letter), and “tgTtt” at the position 29170. Particularly, “tgTtt”, “cgTtt”, “agTtt” and “tgTtt” located at the positions 25402, 26258, 26494 (Figure 4C) and 28235 co-occurred with the NSP15 cleavage motif of four genes (*ORF3a*, *E*, *M* and *N*). In addition, “tgTtt”, “tgTtt”, “ttctT” (the “AAGAA-like” motif on the antisense strand) and “tgTtt” were located at the positions 21566, 21570, 21577 and 21579 (Supplementary Table S1), which are closely linked and flanking the GTTCGT motif of the *S* gene, which merits investigation in the future. The above findings indicated that METTL3 functions in RNA (m6A) methylation of sequences containing the AAACH motif for *ORF3a*, *E*, *M* and *N*, and possibly the “AAGAA-like” motif for *S, ORF6, 7a* and *8*. Finally, we proposed the theoretical arrangement of NSP12-15 and METTL3 in the global RTC structure (Figure 4D) by the integration of information from many aspects, particularly including: 1) identification of NSP15 cleavage sites in our previous study (Li et al., 2021a); 2) discovery of the AAACH motif co-occurred with the NSP15 cleavage motif of four genes; 3) discovery of the association between RNA methylation and NSP15 cleavage; and 4) discovery of the TRS hairpins of eight genes (*S, E, M, N,* and *ORF3a, 6, 7a* and *8*).

By comprehensive analysis of the above results, we constructed a model to answer how the RTC functions in the jumping transcription of CoVs. In this model, the RTC processes double-strand RNAs (dsRNAs) and single-strand RNAs (ssRNAs) in two situations (Figure 4D), respectively. In the first situation, NSP13 unwinds dsRNAs (Yan et al., 2020) to produce ssRNAs(+) or ssRNAs(-), which are processed in two routes. In one route, NSP12 synthesizes RNAs with error correction by NSP14 to produce dsRNAs using unwound ssRNAs(+) or ssRNAs(-) as templates (Knoops et al., 2008). The other route processes ssRNAs(+) or ssRNAs(-), which can be methylated at internal sites and cleaved by NSP15 for jumping transcription. Then, the ssRNAs(+) and ssRNAs(-) are further processed in different ways: most ssRNAs(+) are uncleaved and packaged by the N protein (this is still not clear), while ssRNAs(-) can be uncleaved or cleaved for jumping transcription or degraded, which is regulated by a negative feedback mechanism (Li et al., 2021a). In the second situation, the RTC processes ssRNAs: uncleaved ssRNAs(+) and ssRNAs(-) are used as templates for replication; cleaved ssRNAs(-) are used as templates for transcription. The model can be used to explain the extremely high ratio between sense and antisense reads analyzed in our previous study (Li et al., 2021a) and the experimental result that knockdown of NSP15 by mutation increases the accumulation of viral dsRNA in another previous study (Deng et al., 2017). According to our model, knockdown of NSP15 increases the uncleaved gRNAs(-), which continue to be templates to produce more dsRNAs.

## Conclusion and Discussion

In the present study, we proposed the theoretical arrangement of NSP12-15 and METTL3 in the global RTC structure and constructed a model to answer how the RTC functions in the jumping transcription of CoVs. More importantly, our results reveal the complex associations between RNA methylation, NSP15 cleavage, CoV replication and transcription at the molecular level. Our findings enrich fundamental knowledge in the field of gene expression and its regulation, providing a crucial basis for future studies. NSP12-14 form the main structure of the RTC; NSP7 and NSP8, acting as the cofactors of NSP12, may be also included in the main structure of the RTC (Yan et al., 2020); NSP15 and METTL3 are coupled with the main structure. The results of previous experiments suggest that NSP8 is able to interact with NSP15 (Lianqi et al., 2018). Future research needs to be conducted to determine the structures of NSP12&14, NSP12&15, NSP12&METTL3 and NSP15&METTL3 complexes by Cryo-EM. These local RTC structures can be used to assemble a global RTC structure by protein-protein docking calculation. Our model does not rule out the involvement of other proteins (*e.g.*, ORF8) in the global RTC structure or other proteins in the internal methylation of the “AAGAA-like” motif. Future drug design targeting SARS-CoV-2 needs to consider protein-protein and protein-RNA interactions in the RTC, particularly the structure of NSP15 and the TRS hairpin complex.

## Materials and Methods

The *Betacoronavirus* genus includes five subgenera (*Embecovirus*, *Sarbecovirus*, *Merbecovirus*, *Nobecovirus* and *Hibecovirus*), which were defined as subgroups A, B, C, D and E (Bei et al., 2022). In our previous study (Li et al., 2021b), 1,265 genome sequences of viruses in the *Embecovirus*, *Sarbecovirus*, *Merbecovirus*, *Nobecovirus* subgenera were downloaded from the NCBI Virus database (https://www.ncbi.nlm.nih.gov/labs/virus). Two genome sequences (RefSeq: NC_025217 and GenBank: KY352407) of viruses in the *Hibecovirus* subgenus were also downloaded. Among 1,265 genomes, 292 belongs to *Betacoronavirus* subgroup B (including SARS-CoV and SARS-CoV-2). 1,178, 480 and 194 genome sequences of viruses in the *Alphacoronavirus*, *Gammacoronavirus* and *Deltacoronavirus* genera were downloaded to validate the TRS motifs (Figure 1). Nanopore RNA-seq data was downloaded from the website (https://osf.io/8f6n9/files/) for reanalysis. Data cleaning and quality control were performed using Fastq_clean (Zhang et al., 2014). Statistics and plotting were conducted using the software R v2.15.3 with the Bioconductor packages (Gao et al., 2014). Protein structure data (PDB: 6 × 1B, 7BQ7 and 7CXN) were used to analyze NSP15, NSP10&16 and NSP7&8&12&13, respectively. The structures of NSP12-16 were predicted using trRosetta (Yang et al., 2020). The minimum free energies (MFEs) of hairpins were estimated by RNAeval v2.4.17 with parameters by manual adjustment.

## Data Availability

The datasets presented in this study can be found in online repositories. The names of the repository/repositories and accession number(s) can be found in the article/Supplementary Material.
